# Daily Energy Drink Intake and Arrhythmia Resistant to Direct Current Cardioversion

**DOI:** 10.7759/cureus.83456

**Published:** 2025-05-04

**Authors:** Nawaf Y Aloraini, Anas M Alowaidi, Usamah I Alqarni, Ihab Suliman, Haitham A Alanazi

**Affiliations:** 1 College of Medicine, King Saud Bin Abdulaziz University for Health Sciences, Riyadh, SAU; 2 Cardiology, King Abdulaziz Medical City, King Abdulaziz Cardiac Center, Ministry of National Guard Health Affairs, Riyadh, SAU

**Keywords:** arrhythmia, cardioversions, dc shock, energy drinks, resistant

## Abstract

Supraventricular tachycardia (SVT) is a common arrhythmia, often responsive to pharmacologic intervention or electrical cardioversion. Resistance to direct current cardioversion (DCCV) is uncommon and can be clinically challenging. Lifestyle factors, including dietary stimulant intake, may influence arrhythmia dynamics and treatment responsiveness. We present a case of a 26-year-old female known to have SVT, who presented with six hours of palpitations, with a past medical history of successful cryoablation performed in 2021 for atrial tachycardia originating from tissue near the atrioventricular bundle. Her maintenance therapy included bisoprolol 2.5 mg. Notably, the patient reported regular and excessive consumption of energy drinks, averaging multiple servings daily. The pharmacological intervention failed to achieve rhythm control, necessitating DCCV. Despite appropriate sedation and energy dosing, initial cardioversion attempts were unsuccessful. Subsequent higher-energy shocks ultimately restored sinus rhythm. This case underscores an unusual presentation of cardioversion-resistant SVT in a young adult with prior ablation. The patient’s habitual intake of energy drinks raises concern for potential pro-arrhythmic and electrophysiologic alterations induced by high caffeine and other stimulant content. While caffeine’s arrhythmogenic potential is recognized, its role in altering myocardial excitability and threshold for cardioversion is not well established. This case adds to the limited literature suggesting that excessive stimulant use may contribute to treatment-resistant arrhythmic episodes. Clinicians should be aware of dietary and lifestyle factors, such as energy drink consumption, which may influence the efficacy of electrical cardioversion. Further research is warranted to elucidate the mechanisms by which stimulants impact cardioversion outcomes in patients with SVT.

## Introduction

Supraventricular tachycardia (SVT) is a frequently encountered arrhythmia, with a prevalence of approximately 2.25 per 1,000 individuals and an annual incidence of 35 per 100,000 person-years, occurring more commonly in women [1]. It is often caused by reentrant circuits or abnormal automaticity and may be triggered by heightened sympathetic tone [2]. Caffeine and other stimulants, such as those found in energy drinks, can lower the threshold for arrhythmia by increasing catecholamine release and triggering ectopic activity [1,3]. A rising number of case reports suggest an association between energy drink consumption and serious arrhythmic events, especially in individuals with underlying susceptibility [1,3]. SVT is generally managed with vagal maneuvers, adenosine, and beta-blockers, with catheter ablation reserved for recurrent or pharmacologically resistant cases [2]. Nonetheless, stimulant use has been associated with resistance to conventional therapy and higher recurrence rates [1]. The following case report presents a young female with a past medical history of SVT and prior ablation who developed a recurrent arrhythmic episode that proved resistant to multiple conventional therapies, including direct current cardioversion (DCCV), potentially linked to her regular intake of energy drinks and recent cessation of beta-blocker therapy.

## Case presentation

A 26-year-old female known to have SVT, with a past medical history of successful cryoablation performed in 2021 for atrial tachycardia originating from tissue near the atrioventricular bundle and recurrent episodes of SVT, presented to the emergency department with palpitations persisting for six hours. The palpitations were associated with chest pain and shortness of breath. She denied any history of presyncope, syncope, trauma, slurred speech, fever, cough, vomiting, dysuria, or changes in bowel habits.

Vital signs on presentation were as follows: blood pressure of 120/86 mmHg, heart rate of 206 beats per minute, respiratory rate of 21 breaths per minute, and oxygen saturation (SpO₂) of 99% on room air. On examination, the patient was alert, oriented, and conscious. The cardiopulmonary examination was unremarkable, as was the examination of the abdomen and peripheral vasculature. The patient reported discontinuing bisoprolol 2.5 mg one week before admission. She was an occasional smoker and reported frequent consumption of energy drinks. She had a known allergy to clindamycin.

**Figure 1:**
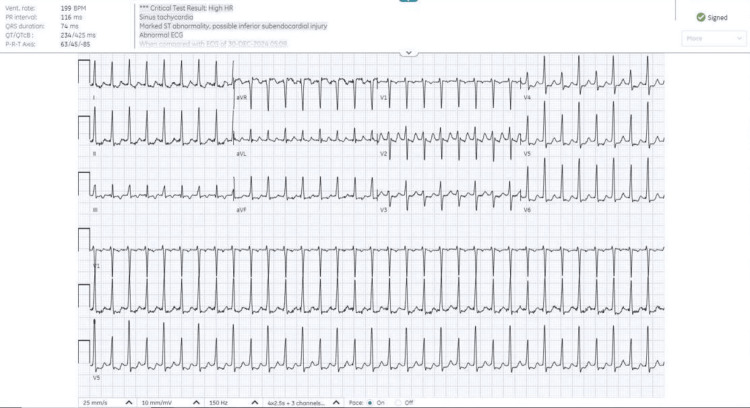
ECG showing a supraventricular tachyarrhythmia.

An electrocardiogram (ECG) (Figure 1) revealed marked ST abnormalities and possible inferior subendocardial injury. Laboratory investigations showed a significantly elevated troponin I level of 210.3 ng/L (reference: <40 ng/L). Other laboratory parameters were within normal limits.

Initial vagal maneuvers (Valsalva) provided transient relief; however, palpitations recurred. The patient was administered metoprolol and adenosine 12 mg and underwent two DCCVs without sustained rhythm control. A total of four cardioversion attempts were made during her emergency department stay, but tachycardia persisted. Following the administration of flecainide, the patient experienced vomiting. She was then transitioned to an amiodarone infusion and transferred to the medical cardiac intensive care unit (MCICU).

**Figure 2:**
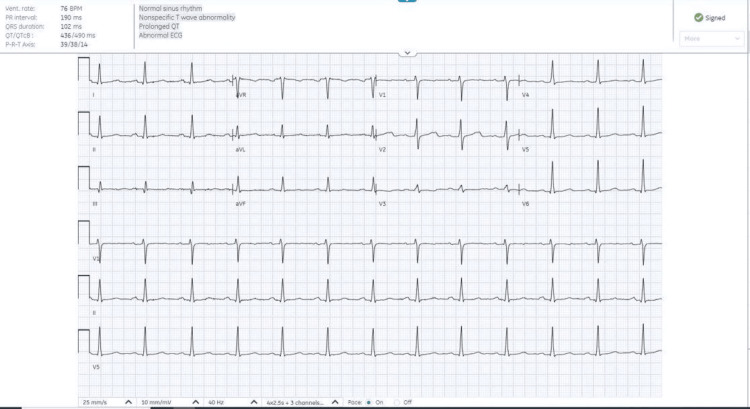
ECG showing normal sinus rhythm and no acute ischemic changes.

After one day, a repeat ECG (Figure 2) demonstrated normal sinus rhythm with nonspecific T-wave abnormalities. The patient remained hemodynamically stable and was discharged home with instructions to resume bisoprolol 2.5 mg once daily and to follow up in the outpatient cardiology clinic.

## Discussion

This case highlights an uncommon presentation of SVT in a young female patient, refractory to multiple conventional treatment modalities. Initial attempts using vagal maneuvers, adenosine, beta-blockers, and multiple DCCVs failed to terminate the arrhythmia. Notably, the patient had a history of SVT status-post ablation in 2021 and had recently discontinued her maintenance bisoprolol 2.5 mg therapy. Her daily consumption of energy drinks is suspected to have played a contributory role in the refractory nature of her arrhythmia.

Energy drinks contain high levels of caffeine and other stimulants, including taurine and guarana, which are known to increase sympathetic activity and heighten the risk of arrhythmias in susceptible individuals [4]. A case report described two healthy adolescents who developed atrial fibrillation after consuming highly caffeinated beverages. Both presented with palpitations and chest discomfort; one required pharmacological intervention with digoxin, while the other reverted to normal sinus rhythm following intravenous fluid administration [4].

The patient’s initial response to a Valsalva maneuver suggests an atrioventricular-nodal reentrant tachycardia. However, recurrence and eventual resistance to both pharmacologic and electrical cardioversion raised concern for a more irritable myocardial substrate. Chronic high catecholamine levels from stimulant intake may have exacerbated the arrhythmogenic potential of this substrate. The requirement of amiodarone infusion and intensive care unit monitoring underscores the severity of her presentation.

A similar case report detailed a young male with chronic energy drink consumption who developed malignant ventricular arrhythmias resistant to initial management [5]. The patient required repeated defibrillations and advanced pharmacologic therapy before conversion to sinus rhythm. While our patient’s arrhythmia was supraventricular, both cases shared notable features: daily stimulant intake, the initial response to non-invasive measures, and subsequent resistance to conventional therapy, including direct current (DC) shock. These parallels support the hypothesis that chronic stimulant exposure alters myocardial excitability and refractoriness, diminishing the efficacy of standard interventions.

In our case, the patient had a history of atrial tachycardia originating near the atrioventricular bundle, status post cryoablation in 2021, indicating a pre-existing conduction abnormality. The recent discontinuation of beta-blockers, along with stimulant intake, may have further destabilized the myocardium and triggered the arrhythmic episode. Similarly, in the comparative case, the absence of structural heart disease alongside an arrhythmogenic presentation suggests acquired electrical instability induced by external stimulants [5]. These findings suggest an interplay between intrinsic cardiac vulnerability and extrinsic triggers in the development of refractory arrhythmias.

Management in both cases followed a stepwise escalation consistent with current guidelines. From vagal maneuvers to advanced pharmacotherapy and ICU care, these cases emphasize the importance of considering lifestyle factors in arrhythmia evaluation. A third report from the Asian Journal of Medicine and Health documented a young man who developed fast atrial fibrillation after consuming three cans of energy drinks, revealing an underlying, previously undiagnosed Wolff-Parkinson-White (WPW) syndrome following cardioversion [6].

Multiple studies have demonstrated a clear association between stimulant use and cardiac arrhythmias. Cocaine use, for example, has been linked to a 61% increased risk of atrial fibrillation, while methamphetamine users have shown a significantly higher incidence of arrhythmias compared to non-users [7]. Additionally, a review of 32 published case reports found that approximately 62.5% of energy drink-related adverse events involved arrhythmias, including both supraventricular and ventricular types [8]. Despite these findings, there remains a notable lack of population-based data specifically examining the relationship between energy drink consumption and SVT, underscoring the need for further research to clarify the electrophysiologic impact of dietary stimulants.

## Conclusions

Our case highlights the need for clinicians to routinely screen for stimulant use, including energy drinks, in patients presenting with arrhythmias. Adherence to maintenance medications such as beta-blockers is also crucial, especially in those with known arrhythmic conditions. This case reinforces the potential for chronic energy drink consumption to exacerbate underlying arrhythmias and lead to treatment-resistant episodes. Detailed history-taking and patient education regarding stimulant use are essential components in preventing recurrent or severe arrhythmic events.
